# Hydrothermal Carbon as Reactive Fillers to Produce Sustainable Biocomposites with Aromatic Bio-Based Epoxy Resins

**DOI:** 10.3390/polym13020240

**Published:** 2021-01-12

**Authors:** Iuliana Bejenari, Roxana Dinu, Sarah Montes, Irina Volf, Alice Mija

**Affiliations:** 1 Institute of Chemistry of Nice, University Côte d’Azur, UMR CNRS 7272, 06108 Nice, France; Juliana.bejenari@tuiasi.ro (I.B.); roxana.condruz@unice.fr (R.D.); 2 Faculty of Chemical Engineering and Environmental Protection, Gheorghe Asachi Technical University of Iasi, 73 Prof. D. Mangeron Street, 700050 Iasi, Romania; iwolf@tuiasi.ro; 3 CIDETEC, Basque Research and Technology Alliance (BRTA), Po. Miramón 196, 20014 Donostia-San Sebastián, Spain; smontes@cidetec.es

**Keywords:** hydrothermal carbon, bio-based epoxy resins, sustainable biocomposites, natural materials, waste valorization

## Abstract

Thiswork is focused on the development of sustainable biocomposites based on epoxy bioresin reinforced with a natural porous material (hydrochar, HC) that is the product of spruce bark wastes subjected to hydrothermal decomposition. To identify the influence of hydrochar as a reinforcing material on the designed composites, seven formulations were prepared and tested. An aromatic epoxy monomer derived from wood biomass was used to generate the polymeric matrix, and the formulations were prepared varying the filler concentration from 0 to 30 wt %. The reactivity of these formulations, together with the structural, thermal, and mechanical properties of bio-based resin and biocomposites, are investigated. Surprisingly, the reactivity study performed by differential scanning calorimetry (DSC) revealed that HC has a strong impact on polymerization, leading to an important increase in reaction enthalpy and to a decrease of temperature range. The Fourier Transform Infrared Spectroscopy (FT-IR) investigations confirmed the chemical bonding between the resin and the HC, while the dynamic mechanical analysis (DMA) showed increased values of crosslink density and of storage moduli in the biocomposites products compared to the neat bioresin. Thermogravimetric analysis (TGA) points out that the addition of hydrochar led to an improvement of the thermal stability of the biocomposites compared with the neat resorcinol diglycidyl ether (RDGE)-based resin (T_5%_ = 337 °C) by ≈2–7 °C. Significantly, the biocomposites with 15–20 wt % hydrochar showed a higher stiffness value compared to neat epoxy resin, 92SD vs. 82SD, respectively.

## 1. Introduction

One of the current global problems is finding sustainable solutions to replace fossil-based polymers, composites, and materials to stop the very seriously damaged already produced on the environment. Those kinds of materials are considered non-renewable resources that need millions of years to form again. The growing needs of a world in continuous progress and development have contributed to the fossil resources becoming more and more limited and their price unpredictable. The main disadvantage of using fossil fuels is the elimination of carbon dioxide in the atmosphere, which leads to environmental damage and ultimately contributes to global warming [1]. To reduce these effects, global targets have been set for increasing the use of renewable resources by chemical companies. In addition, a number of new regulations based on European directives [2,3,4,5] on reducing the volume of waste and pollutants have led to the development of green chemistry and the search for new alternatives to replace the large number of substances classified as carcinogenic, mutagenic, and reprotoxic abundantly used in daily life. Nowadays, due to their versatility in tailoring their ultimate physico-chemical and mechanical properties as well as their final performances generated by their high crosslinked structures, thermosetting polymeric materials are widely used in the engineering fields [6]. Among the main thermoset systems used in fields such as composites, adhesives, coatings, civil engineering, automotive sector, and electrical materials are the epoxy resins [7,8,9]. They are usually synthesized by the reaction of polyols, polyphenols, or other active compounds of hydrogen with epichlorohydrin under basic conditions [10]. One of the main constituents of the thermoset epoxy resins is known as bisphenol A (BPA), which is obtained from petroleum resources and has been classified as a carcinogen, mutagen and reprotoxic (CMR R3) substance [11,12]. The imminent need to limit the use of fossil resources, to reduce the environmental pollution as well as address the recent awareness of BPA toxicity on the human body, has led to the development of intensive scientific studies to find new bio-based and regenerable alternatives for the development of green epoxy thermoset materials [13].

Thermoset-based composites are materials that have had an innovative development in recent years. According to the reported studies, among the optimal fillers used for polymeric (nano)composites with superior mechanical, electrical, and chemical properties are the carbon nanotubes and grapheme [14,15,16,17,18]. Unfortunately, this type of material leads to a high production cost. In contrast, it was found that hydrochar and biochar represent a new source of bio-carbon fillers for composites materials instead of fossil fuel extracted additives [19]. These types of products are carbonaceous materials obtained through thermomechanical conversions as pyrolysis [20], or the hydrothermal carbonization [21] of different sources of biomass. Hydrothermal carbonization is a biomass conversion technique that is achieved through a series of reactions such as hydrolysis, dehydration, decarboxylation, aromatization, and re-condensation [22]. Compared to other conversion methods, the main advantage of this process is the use of wet biomass, thus leading to a significant reduction in the cost of production, because a pre-drying treatment of biomass is not necessary [23]. Hydrothermal carbonization is considered a green process, being non-toxic, cheap, environmentally friendly, and easy to be applied [24]. To the best of our knowledge, only a reduced number of studies have been conducted on the utilization of hydrochar as filler in the production of polymeric composites [25,26]. For example, Khan et al. [27] studied the mechanical and electrical behavior of composites developed from an commercial epoxy resin as matrix reinforced with two types of biochar, pristine and heat-treated biochar, made from maple wood. The authors compared the properties of the designed materials with those of composites produced from commercial multiwalled carbon nanotubes, proving that the biochar can be a low-cost, ecofriendly, renewable alternative for the commercial carbon fillers. Giorcelli et al. [28] designed epoxy-based composites reinforced with biochar and biochar heat treated, both derived from maple tree, investigating the influence of the filler morphology on the mechanical properties of the final composite materials. Other studies have reported that not only the morphology of the filler influences the properties of the final composites, but also the quantity and nature of the raw material from which the biochar is obtained can influence the performance of the final materials [19,29].

The main objective of this work is the design of new eco-friendly composite materials based on bio-renewable raw materials combined with hydrochar (HC). The bio-based thermoset matrix was obtained by the polymerization of an aromatic epoxy compound derived from wood biomass such as resorcinol diglycidyl ether. As filler, an HC obtained by the hydrothermal conversion of spruce bark was used. The aims of this work are (i) to establish the optimal bio-based epoxy resin/filler formulation, (ii) to investigate the influence of the hydrochar content on the crosslinking reactivity, and (iii) to evaluate of the effect of the HC on the physico-chemical and mechanical properties of the composite materials. To reach these objectives, the reactivity studies were followed by DSC and FT-IR. The morphological characteristics of the prepared composites were analyzed by SEM, and the thermomechanical properties were investigated by differential scanning calorimetry (DSC), thermogravimetric analysis (TGA), dynamic mechanical analysis (DMA), tensile tests, and Shore tests.

## 2. Experimental Section

### 2.1. Materials

The thermoset resins were developed by the polymerization of resorcinol diglycidyl ether (RDGE), which was initiated by N,N-dimethylbenzylamine (BDMA) as catalyst and 2,4,6-tris(dimethylaminomethyl)phenol (DMP-30) as accelerator. The compounds were purchased from Sigma-Aldrich (France) and used as received. The obtained bio-based resins were reinforced with hydrochar (HC) to develop biocomposites. The hydrochar (HC) was obtained by a hydrothermal carbonization process and appears as a brown powder with a particles size distribution of 400 µm.

#### Hydrothermal Carbonization and Characterization of HC

To produce HC in large quantities, a spruce bark powder was subjected to hydrothermal carbonization in a 2 L stainless steel autoclave. The process was carried out in a closed aqueous system under mild temperature (280 °C) with self-generated pressure under a residence time of 1 h. At the end of the process, the autoclave was cooled to the room temperature. The obtained products were a mixture of a solid phase, the hydrochar, and a liquid phase, the biocrude oil. The mixture was filtered, and the hydrochar washed several times with distilled water and dried in an oven at 105 °C for 24 h.

To determine the HC’s particles size distribution, an Anton Paar PSA 1190 LD apparatus in solid phase with air pressure at 2500 mbar was used. The distribution of the HC particles was in the range of 0.1‒500 μm, with a major size distribution around 400 μm (Table S1). The elemental analysis of the synthesized HC was done using EDX analysis showing that the principal component is represented by carbon. The elemental content of HC is 71.61% C, 21.11% O, 5.20% H, 1.44% Ca, 0.38% Cu, and 0.26% Zn, while the wood bark is characterized by the following composition: 58.26% C, 33.15% O, 5.84%, H, 0.05% Mg, 0.01% Al, 0.01% Si, 0.04% P, 0.05% S, 0.01% Cl, 0.33% K, 1.59% Ca, 0.02% Mn, 0.38% Cu, and 0.27% Zn. During the hydrothermal carbonization of the spruce wood, the deoxygenation, dehydration, and decarboxylation reactions took place, which favored the increase of the carbon content and the decrease of the oxygen content [30]. No traces of magnesium, silicon, sulfur, potassium, or aluminum were found.

### 2.2. Composites Preparation

Prior to the composites’ preparation, the HC was dried in oven at 105 °C for 24 h. For a better dispersion of the filler in epoxy matrix, the RDGE was heated on a hot plate at 50 °C until its viscosity decreases. In the liquid RDGE, the proper weight percentage of filler was added and mixed to homogenize. The weight percentages of HC into the mixture formulations were 1, 5, 10, 15, 20 and 30 wt %. To the RDGE + HC mixture, a catalytic combination of 5 wt % of BDMA and DMP-30 was added. Each formulation was transferred into silicone molds and thereafter cured and post-cured. Firstly, the mixtures were cured in oven at 80 °C for 1 h and then at 130 °C for 30 min. For a complete crosslinking, the bio-based composites were post-cured during 30 min at 160 °C. Each sample is assigned a code representing the composition of the formulation and the mass percentage of the components. “R” represents the RDGE, with “BD” represents the mixture between BDMA and DMP-30, and “HC” is the abbreviation for hydrochar. The numbers given in the abbreviations correspond to their mass percentage; e.g., “R94-BD5-HC1” acronym corresponds to the formulation containing 94 wt % RDGE, 5 wt % BDMA+DMP-30 (2.5 + 2.5 wt %), and 1 wt % of HC.

### 2.3. Experimental Techniques

#### 2.3.1. Differential Scanning Calorimetry (DSC)

The influence of the HC on the epoxy crosslinking reactivity was analyzed using a DSC 3 Mettler Toledo apparatus operated by STARe Software. Freshly prepared samples of uncured formulations of 5–7 mg were placed into 40 µL aluminum pans and studied under dynamic conditions between 25 and 250 °C at a heating rate of 10 °C·min^−1^. The reactions’ enthalpy values of the analyzed mixtures with HC were normalized to the mass of the resin in the analyzed compositions. The DSC method was also used for the investigation of the secondary transitions of the bio-based composites (glass transitions, T_g_). Crosslinked samples with mass between 5 and 7 mg were placed into 40 µL Al crucibles and scanned under two heating/cooling cycles from 25 to 180 °C at a 10 °C·min^−1^ heating rate. The T_g_ values were measured as the inflection point of the DSC curves in the second heating scan.

#### 2.3.2. Fourier Transform Infrared Spectroscopy (FT-IR)

The recording of IR spectra was performed using a Nicolet iS50 Fourier Transform Infrared Spectroscopy (FT-IR) spectrometer equipped with a GladiATR (PIKE Technologies, Inc., Madison, WI, USA) single diamond attenuated total reflectance. The FT-IR spectra of all the samples were collected in the range 4000–600 cm^−1^ at a resolution of 4 cm^−1^ and 32 scans. This technique was applied to analyze the structure of raw materials, fresh mixtures, and of the cured final resin and composites.

#### 2.3.3. Thermogravimetric Analysis (TGA)

A TGA 2 Mettler Toledo apparatus was used to study the thermal stability of the materials. For each experiment, approximately 10 mg of the crosslinked sample were placed into a 70 μL alumina pans and thermally treated from 25 to 1000 °C at a heating rate of 10 °C·min^−1^ in an oxidative atmosphere (air) with a flow rate of 50 mL·min^−1^.

#### 2.3.4. Dynamic Mechanical Analysis (DMA)

The influence of the HC on the mechanical properties such as storage modulus (E’), loss modulus (E”), and damping factor (tan δ) of the prepared resin and composites was analyzed by DMA. The DMA tests were accomplished using a Mettler-Toledo DMA 1 device equipped with a three-point bending clamp. The mechanical behavior of the rectangular samples (48 × 8 × 4 mm^3^) was measured under nitrogen atmosphere in a temperature-scanning mode ranged from −30 to 200 °C at a heating rate of 3 °C·min^−1^, an oscillatory frequency of 1.0 Hz, and an amplitude of 20 µm.

According to ASTM D7028-07 and AITM 1-0003 standards, the DMA measurements were used to determine three values of the glass transition region such as T_g-onset_ (related to the drop of storage modulus), T_g-loss_ (maximum temperature on the loss modulus), and the tan δ (maximum temperature of the damping factor). Using the DMA results, we calculated the crosslink density (ν, mmol∙cm^−3^) of the resin and biocomposites. According to Flory’s theory [31], the value of the E’ in the rubber region permits the calculation of the crosslink density using the equation:

$$\upsilon =\frac{E^{\prime }}{3\mathrm{RT}}$$

where E′ is the storage modulus of the thermoset in the rubbery plateau region at T_g_ + 80 °C (MPa), R represents the gas constant, and T is the absolute temperature (K).

#### 2.3.5. Tensile Testing

According with the ISO 527-1 and ASTM D638-08 standards, the mechanical properties such as tensile strength, elongation at break, and Young’s modulus of the biocomposites were tested. The developed materials were analyzed using a mechanical universal testing machine Instron, model 3365, controlled by BlueHill Lite software developed by Instron (Norwood, MA, USA). Four rectangular samples with the dimension of 75 × 10 × 4 mm^3^ were examined applying a crosshead speed of 10 mm·min^−1^, and the obtained data were averaged for a better accuracy of the results.

#### 2.3.6. Shore Hardness Tests

To analyze the stiffness of produced materials, a Zwick Roell 3116 Hardness Tester was used. The samples were tested with a Shore D device in accordance with the ISO 7619-1, ASTM D2240, and ISO 868 standards. The load force applied to the specimens was ≈50 N ± 0.5 N, and the hardness values were read at the firm contact between the presser foot and the tested materials. The measurement errors were reduced by testing five different specimens from each formulation.

#### 2.3.7. Scanning Electron Microscopy (SEM)

To investigate the surface morphology of the developed biocomposites, fresh fractures of cured materials were investigated by scanning electron microscopy (SEM). The samples were mounted on a SEM stub, coated with platinum, and then observed using a SEM Tescan Vega 3 XMU at an acceleration voltage of 5 kV.

#### 2.3.8. Density

To determine the materials density, samples with a geometry of 50 × 8 × 5 mm^3^ for each formulation were weighed, and their volume was calculated. The materials’ density was calculated as the ratio of mass to volume, on three samples, considering the average value.

#### 2.3.9. Water Absorption

The determination of the water absorption capacity was performed by the gravimetric method according to ISO 62:2008. Rectangular samples with the size of 13 × 8 × 4 mm^3^ were immersed in distilled water at room temperature (25 °C) for 30 days. The absorption percentage of the analyzed samples was calculated by applying the following equation:

$$\mathrm{Water}\;\mathrm{absorption}\;\%=\frac{w_{f}-w_{i}}{w_{i}}\times 100$$

where w_i_ represents the conditioned mass and w_f_ is wet mass of the samples. At every 24 h, the samples were removed from water, gently dried with blotting paper, and their wet mass was measured.

#### 2.3.10. Solvent Stability

The solvent stability of materials was determined by using seven solvents: methanol (MeOH), acetone (Ac), toluene (T), N, N-dimethylformamide (DMF), dimethyl sulfoxide (DMSO), acetonitrile (AcN), and chloroform (CHCl_3_). Rectangular samples (13 × 8 × 4 mm^3^) from each formulation were weighed to establish their conditioned weight (w_f_); thereafter, the samples were immersed in the selected solvents for 30 days. Every day, the immersed samples were removed form solvents, dried with filter paper to eliminate the solvent excess, and then, their wet weight (w_i_) was measured. The solvent stability of the bio-based materials was calculated using the similar equation as for the water absorption test.

## 3. Results and Discussion

### 3.1. Reactivity Study of Crosslinking in the Presence of HC

#### DSC Investigation

**Figure 1:**
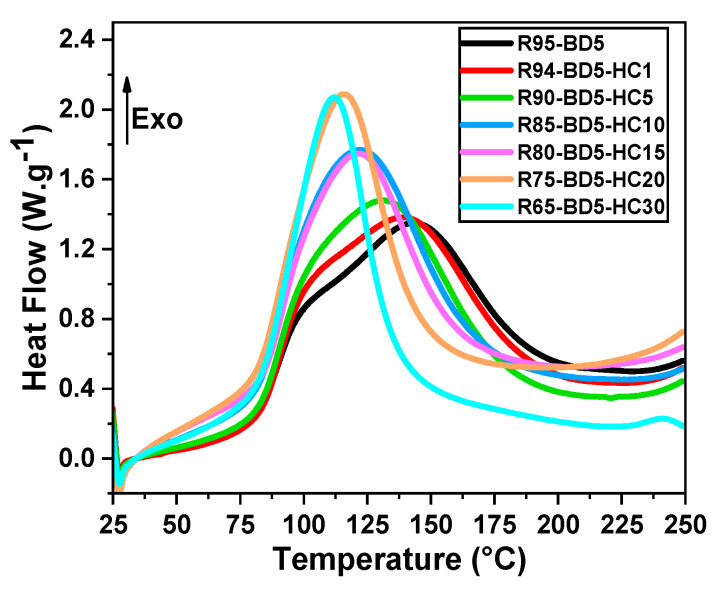
Dynamic differential scanning calorimetry (DSC) curves during heating at 10 °C/min of the neat resin and resorcinol diglycidyl ether (RDGE)/BD/hydrochar (HC) formulations.

To establish the thermodynamic parameters of RDGE crosslinking in the presence of HC and moreover to study the HC influence on the thermal curing, the prepared formulations were investigated by dynamic DSC. Figure 1 shows the evolution of heat flux vs. temperature for the neat RDGE resin and for its mixtures with different amounts (1‒30 wt %) of HC. Table S4 summarizes the obtained data in terms of reaction enthalpy and temperature range for the curing reactions.

We can observe in Figure 1 that the crosslinking reactions occur as well-defined exothermic events, which are significantly affected by the addition of the biocarbon filler. In this figure, we can constate that the neat resin shape of the reaction changed dramatically with the increasing of the filler content. Starting with 5% of HC, the reaction range decreases, while the reaction enthalpy increased. The onset of curing for the neat resin takes place at a temperature of about 66 °C, and those of RGDE-HC mixtures are shifted to lower temperatures, decreasing with around 3–13 °C with the increasing of HC content. For the neat resin, the completion of curing occurs at high temperature (≈230 °C), while that of composite with 30 wt % HC at around 170 °C.

The area under the exothermic peaks was integrated to obtain the heat of cure for each formulation and normalized to the mass of the resin. Following the data given in Table S4, we can deduce that the presence of HC leads to an important increase in the reaction enthalpy and to a decrease in the maximum reaction temperature (T_max_). These two results are the sign that the HC is contributing and participating in the chemical bonding, which is also having a catalytic effect on the initiation of the cure reaction. The maximum temperature of curing (T_max_) of the neat resins is at around 141 °C, while with the addition of the HC in the mixtures, the T_max_ value decreases to 112 °C with the 30% HC content. For this formulation, R65-BD5-HC30, the cure is accompanied by the highest enthalpy of reaction of around 512 J.g^−1^.

In conclusion, the bio-carbon has a strong effect on RDGE crosslinking, acting as a catalyst by increasing the system reactivity and decreasing the crosslinking temperature range, as well as to a shift of the T_max_ to lower temperatures.

### 3.2. Fourier Transform Infrared Spectroscopy (FT-IR)

#### 3.2.1. FT-IR Analysis of HC

Functional groups that are present in hydrochar (Figure S1) were identified by a FT-IR screening and were correlated with the reported studies [24,32,33,34]. Figure S1 shows the spectra of hydrochar with characteristic peaks at around 3000–3500 cm^−1^ (O-H), 2922 cm^−1^ (C-H), 1693 cm^−1^ (C=O), 1599 cm^−1^ (C=C), 1446 cm^−1^ (C-H), 1203 cm^−1^ (C-O), 1027 cm^−1^ (C-O), and 870–750 cm^−1^ (C-H). The origin of the functional groups is attributed to the degradation products of the spruce bark main compounds: hemicelluloses, cellulose, and lignin, resulting after several chemical reactions: hydrolysis, dehydration, decarboxylation, aromatization, and recondensation, which took place during hydrothermal carbonization. During hydrolysis, the hemicelluloses were transformed into pentose, the celluloses were transformed into oligosaccharides or even hexoses, and the lignins were transformed into polyphenols with different molecular weight. The dehydration reaction leads to the elimination of water from the feedstock matrix. Due to the appearance of dehydration and decarboxylation reactions, the double bonds (C=O and C=C) could be replaced by single bonds such as carboxyl and hydroxyl groups. The final reaction of recondensation of degraded byproducts leads to the formation of hydrochar [35].

#### 3.2.2. Reactivity Study by FT-IR

To evaluate the structural modifications occurring during curing reaction, FT-IR analyses in attenuated total reflection mode (ATR) of raw materials, reactive mixtures, and crosslinked materials were performed. Table S5 shows the main functional groups assigned to each component compound of the crosslinking formulation. For the RDGE structure, the low intensity band at 3062 cm^−1^ is assigned to the characteristic stretching vibration of the C-H bond of the methylene groups in the aromatic rings. The stronger peaks at 1590 cm^−1^ and 1491 cm^−1^ are attributed to the stretching vibration of the C=C and C-C bond of aromatic rings. Absorption peaks with a medium to moderate intensity corresponding to the wavenumbers of 1260, 902, and 842 cm^−1^ are assigned to the oxirane rings. The peaks that are identified in the range of 800–500 cm^−1^ correspond to the out-of-plane deformation of C-H bonds. For the curing agent (BDMA) and accelerator (DMP-30), the absorption peaks appearing in the range of 3085−2763 cm^−1^ are assigned to the asymmetrical and symmetrical stretching vibration of the C-H bond of the –CH_2_ and –CH_3_ groups. The stretching deformations of the C-H bond of the –CH_3_ and –CH_2_ groups are characteristic for the wavenumbers between 1354 and 1495 cm^−1^. The low absorption intensity at 1611 cm^−1^ corresponds to the stretching vibration of the C=C skeleton in the benzene ring.

The medium absorption peaks in the range 900–600 cm^−1^ are attributed to the out-of-plane deformations of the =CH bonds from the benzene rings. After the FT-IR analysis of each compound, the evolution of spectra corresponding to the reactive mixture and crosslinked materials were analyzed. These evolutions are represented by the appearance or disappearance of some absorption peaks, as well as a possible decrease or increase of the intensity of the compounds’ absorption peaks.

**Figure 2:**
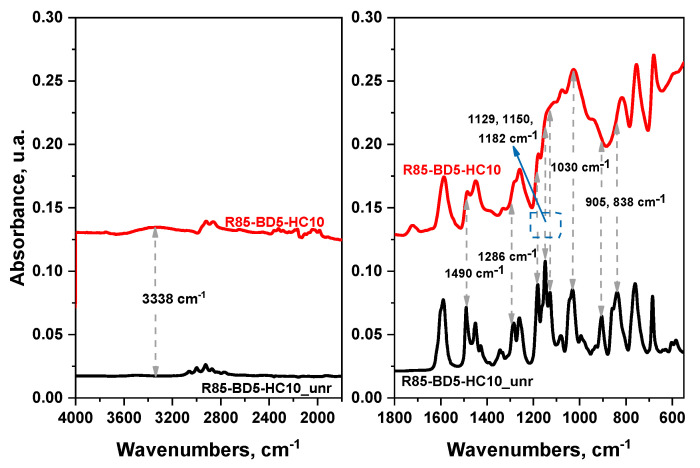
Fourier Transform Infrared Spectroscopy (FT-IR spectra) of unreacted mixture and crosslinked resin for the formulation with 10% HC.

Figure S3 shows that the intensity of the peaks in the range of 500–1700 cm^−1^ are decreasing after crosslinking, which is due to the epoxy groups consumption during the reaction. Indeed, Figure 2 shows the disappearance of the peaks with the maximum absorption at 905 and 838 cm^−1^ and the appearance of a weak band at 3394 cm^−1^, corresponding to the opening of the epoxy ring during crosslinking. These results pointed out that the epoxy functional groups were completely reacted.

In addition, the presence of a large and weak band at 3338 cm^−1^ could be explained by possible changes that occurred in the hydrogen bonding network of HC in the presence of epoxy resin, leading to a possible opening of the epoxy ring. We can highlight that the region of ether bands at 1129, 1150, and 1182 cm^−1^ is transformed after curing on a large envelop absorption with an intense peak at 1030 cm^−1^. This result is probably due to the higher content on C-O-C bonds formed after reactions. Partly, a part of these bonds is formed during the RDGE polymerization generating the polyether structure. At the same time, another part of the C-O-C bonds can be created during the etherification reactions that could occur between the epoxy rings and the -OH functions from HC. After the polymerization reaction, the peak at 1490 cm^−1^ with a medium intensity corresponding to an asymmetrical and symmetrical stretching deformation of the C-H bond from BDMA has disappeared as well as that at 1286 cm^−1^ attributed to the symmetrical stretching vibration of the epoxy C-O bond.

### 3.3. Physico-Chemical and Mechanical Characterization of HC Biocomposites

#### 3.3.1. Thermogravimetric Analysis

**Figure 3:**
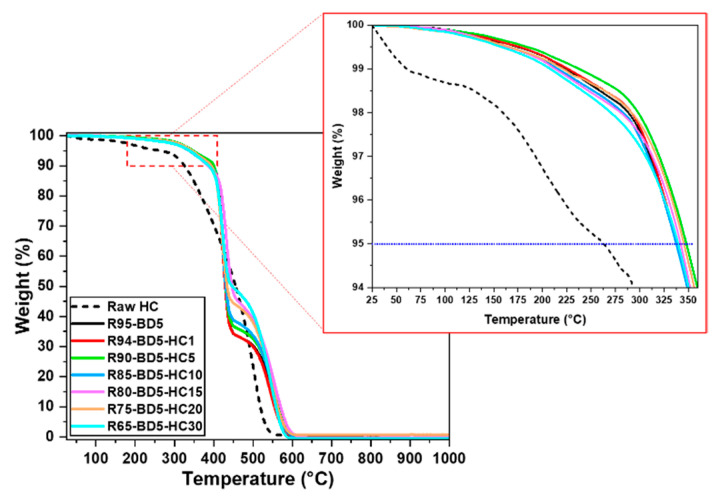
Thermogravimetric analysis (TGA) thermograms of bio-based cured samples.

The TGA analysis of HC-based biocomposites is presented in Figure 3 and Figure S4, respectively. The thermal stability of the raw HC was also analyzed.

The initial weight decrease (≈5 wt %) of the raw HC takes place between 35 and 250 °C and can be attributed to the gradual moisture loss from the sample. From the DTG curve (Figure S4), it can be observed that the main HC decomposition step appears as a large peak with a shoulder ranged between 270 and 425 °C. The weight loss associated to this shoulder is ≈33% and is characteristic to the hemicellulose degradation, while the thermal degradation of cellulose and lignin occurs from 425 to 600 °C (≈62 wt %) [36].

From Figure 3, we can observe that the thermal degradations of the RDGE resin and composites occur in two temperature-dependent stages. The first step represents the major thermal decomposition stage for all the samples, which ranged between 280 and 480 °C and is associated with the thermolysis phenomenon. In this stage, it can be noticed that the addition of the HC filler in the RDGE resins decreases the mass loss of the composites by about 3–10%. For example, the mass loss of the RDGE resin in the first step is ≈67 wt %, while the composites with 30 wt % HC has a mass loss in this stage around 57%. The second decomposition stage of thermal–oxidation and carbonization occurs between 480 and 660 °C. In this step, the maximum decomposition occurs at ≈550 °C and the addition of HC increased the percentage of mass lost at this stage by about 10% compared to the neat resin.

According with the obtained data, the bio-based thermosets resin and composites reveal a very good thermal stability. The degradation temperature of the cured bio-based resin and composites was considered at 5% of the mass loss of materials (T_5%_), the obtained values being summarized in Table 1. From the TGA analysis, we can see that the thermal stability of the RDGE resin (T_5%_ = 337 °C) was improved by ≈2–7 °C by HC reinforcement. The thermal stability of the developed materials was also analyzed by the statistic heat-resistant index (T_s_) calculated by equation [37,38]:

$$T_{s}=0.49\left[T_{5\%}+0.6\left(T_{30\%}-T_{5\%}\right)\right]$$

where T_5%_ is the temperature at which the samples lose 5% of their mass and T_30%_ represents the temperature at which the materials lose ≈30% of their mass. This factor gives the physical heat tolerance limit temperature, and the calculated T_s_ values are presented in Table 1. These values reveal that the developed materials have similar thermal stabilities. The resins reinforced with HC exhibit a very good thermal stability, losing 5 wt % of their mass at temperatures above 340 °C.

#### 3.3.2. Dynamic Mechanical Analysis

**Figure 4:**
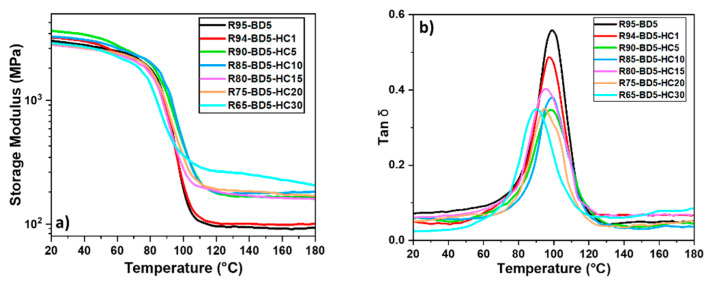
Dynamic mechanical analysis (DMA) analyses of resin and their composites: (**a**) evolution of storage modulus (E′) in function of temperature, and (**b**) the damping factor (tan δ) vs. temperature.

DMA analyses of the bio-based composites were performed to study their viscoelastic behavior represented by the variation of the storage modulus, the loss modulus, and the damping factor as temperature functions in order to understand the interfacial bond between the resin matrix and the HC. Figure 4a reveals the variation of the storage modulus vs. temperature of the bio-based samples, which indicates the stiffness of the materials. Figure 4a illustrates the evolution of three viscoelastic regions: the glassy region followed by the transition from the glassy to rubbery state, and the third region depicted by the rubbery plateau. In the glassy region, at ≈25 °C, the R95-BD5 resin presents a storage modulus value of ≈3 GPa. With the addition of 1, 5 or 10 wt % HC, it can be seen an increase of E’ values above 3 GPa, the highest value being obtained for the composites with 5 wt % HC, E’ ≈3.6 GPa. For the composites with 15, 20 or 30 wt % HC, a decrease of the E’ values can be observed in the glassy plateau, reaching ≈2.7–2.8 GPa. As previously demonstrated by the DSC study, HC is not only a filler, because it acts as a reaction initiator and as a coreactant in the polymerization mechanism. So, due to this double role of the HC, chemical and physical, the difference in the thermomechanical behavior of the systems is due to the chemical bonding between the epoxy and the hydrochar, generating different kind of materials with different polymeric networks.

The elastic response of the bio-based materials in the rubber region was strongly influenced by the addition of HC, its value increasing with the filler amount. The storage modulus curve gives information about the crosslinking density of the material: the smaller the drop interval between the glassy and the rubber region, the higher the crosslink density. In general, it is known that the fillers presence in thermoset composites has as an effect a steric disruption of chemical connectivity, thus decreasing the crosslink density of the obtained materials. Based on Figure 4a, it can be seen that the biomaterials with HC have a smaller drop between the glassy and rubbery plateau, so they have an increased crosslink density value. This result shows again the HC contribution in the crosslinking mechanism, leading to the development of more compact networks. A similar trend was obtained by the calculated values of the crosslink densities based on Flory’s theory (Table 1).

The calculated value of the resin crosslink density is around 8.23 mmol∙cm^−3^. By the addition of HC, this value increased 2–3 times in the following series: R94-BD5-HC1 < R80-BD5-HC15 < R90-BD5-HC5 < R75-BD5-HC20 < R85-BD5-HC10 < R65-BD5-HC30. An analogous influence of the biochar addition on the viscoelastic behavior of the polymeric resin was reported by Zhang et al. [39] who studied the effect of activated biochar on the thermomechanical properties of microcrystalline cellulose/polylactic acid composites.

Figure 4b displays the tan δ curves of the elaborated materials. This factor is a damping property calculated by the ratio between E” and E’, showing the balance between the elastic and viscous phases of polymeric system. A typical peak was observed in the damping factor curve of the RD95-BD5 system exhibiting a well-defined relaxation, reaching the maximum at about 99 °C. The addition of HC in the systems caused a decrease in tan δ peak with about 1 to 10 °C (in function of the HC amount), indicating an improvement of bio-based materials plasticity. The degree of rigidity of the materials is given by their crosslink density. In addition to the crosslinking density already discussed from the drop interval between the glassy and the rubber region or calculated by Flory’s theory, this parameter can also be deduced with the help of the damping factor. Thereby, the higher the intensity of the tan δ peak, the lower the crosslink density of the analyzed material. Studying the damping factor curves (Figure 4b), it can be seen that the addition of HC decreases significantly the peak intensity, the tan δ of the material with 30 wt % HC being reduced by half compared to that of epoxy resin. The trend of the crosslinking density obtained by analyzing the intensity of the tan δ peaks is in accordance with the values already discussed and presented in Table 1. The glass transition region of the materials was also determined from the DMA results using two methods: the onset of the storage modulus drop (T_g-onset_) and the peak of the maximum of the loss modulus (T_g-loss_). Thereafter, the glass transitions obtained by DSC and DMA were compared, the acquired values being presented in Table 1. Given that the T_g_ by DSC is measured under no mechanical stress, while the transitions evaluated by DMA are measured under mechanical stress at a given frequency, the values obtained by the two methods can be quite different. Nevertheless, from Table 1, it can be seen that the tan δ and T_g_ values obtained by DMA and DSC show the same trend: the transition region decreases with increasing HC amount.

The rigidity of the biomaterials was analyzed also by Shore D hardness tests. As given in Table 1, it was determined that the hardness of the epoxy resin is about 82SD, while the highest stiffness was generated by the addition of 15 and 20 wt % hydrochar, obtaining values of ≈92SD. Increasing the HC concentration leads to an increase in materials’ hardness, obtaining values between 82 and 92SD. According to the Shore scale, these values are included in the category of extra hard materials.

So, the opposite effect of T_g_ decrease and of Shore hardness increase shows that the HC contribution is dual, acting both as a filler and as a reactant in the polymerization reaction. This leads to the development of new, more rigid and plastic materials, the brittle effect of neat thermoset resins being diminished.

The densities of HC-reinforced composite and of neat epoxy resin were also calculated and are given in Table 1. We can notice that the addition of 1 wt % HC leads to an increase in the composites’ density. From another point of view, it was found that with the increase of HC ratio in the material composition, the density of the developed composites decreases. This result can be explained by the fact that the HC particles are light and increasing their ratio will cause them to occupy a larger volume in materials. In conclusion, the results of viscoelastic behavior showed that the addition of the proper amount of HC lead to an improvement of the thermomechanical properties of the RD95-BD5 thermoset resin.

#### 3.3.3. Tensile Testing

The influence of the HC addition on the epoxy polymeric matrix was also investigated by the tensile test. The tensile stress–strain tensile behavior of the developed thermoset bio-based materials is displayed in Figure S5.

The curve of neat epoxy resins is also reported for comparison. Based on the obtained data, the presence of very good mechanical properties of the RDGE95-BD5 polymeric resin can be seen, with a considerable tensile stress of approximately 65 MPa together with an important elongation at break (≈7%). The addition of the HC particles in the epoxy matrix decreases the elongation at break and the tensile strength of the obtained biomaterials. According to reported studies [27,28,29,36], several factors can affect the properties of the polymeric materials reinforced with biochar such as the filler concentration in composite, the quality of the feedstock, the process parameters, and also the distribution of the filler in the matrix. Figure S5 shows that increasing the percentage of filler decreases both the stress and strain of the resulting materials. Giorcelli et al. [28] used different percentages (1, 2, 4 and 20 wt %) of biochar derived from maple as an epoxy resin filler, analyzing the variation of the filler’s percentages on the properties of composites. The best mechanical properties were obtained by reinforcing the epoxy resin with 2% biochar, the mechanical behavior of composites being modified from brittle to ductile. The addition of higher biochar content (20%) led to a significant decrease in the mechanical properties of the composites. A similar influence of the biochar percentage used in the composites development was also reported by Khan et al. [27].

The stiffness of the materials, named also as Young’s modulus, is defined by the relationship between stress and strain in the linear elasticity regime of a uniaxial deformation, and the obtained values for the thermoset bio-based systems are tabulated in Table S6. As in the case of stress and strain results, the values of the Young’s modulus for composites filled with HC powder are slightly decreased. The stiffness of the epoxy resin is about 1.8 GPa, while the addition of 5 wt % HC start to decrease this value up to approximately 1.73 GPa. In addition, it can be observed that the increase of the HC amount added in the epoxy resin leads to the progressive decrease of the Young’s modulus value, reaching about 1.4 GPa for the system with 20 wt % biochar. The resilience modulus was also determined, and the obtained values are given in Table S6.

The resilience represents the ability of a material to absorb energy when it is deformed elastically and to release that energy upon discharge without permanent deformation resulting. The modulus of resilience was calculated as the area under the stress–strain curve up to the elastic limit. The epoxy resin shows a very high resilience obtaining values of about 217 MJ/m^−3^. The addition of HC considerably affected this parameter, its value decreasing with about 89–96% as a function of the HC concentration. Therefore, the HC nature and its use in large quantities, but especially its ability to participate in network crosslinking generating new linkages, can led to these mechanical behavior variations.

#### 3.3.4. Morphology of the Bio-Based Polymeric Composites

**Figure 5:**
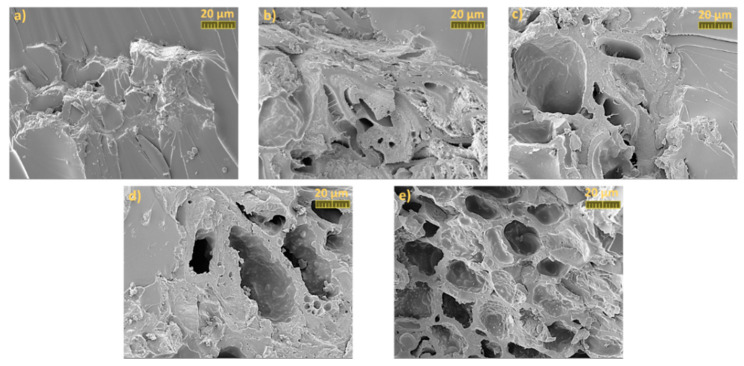
SEM images of epoxy composites filled with (**a**) 1 wt % HC, (**b**) 5 wt % HC, (**c**) 10 wt % HC, (**d**) 15 wt % HC, and (**e**) 20 wt %.

The dispersion but also the adhesion of the HC particles in the epoxy bio-based thermoset resin was investigated by Scanning Electron Microscopy (SEM). Figure 5 show the SEM pictures of the tensile testing fracture surfaces of the epoxy/HC composites at 20 µm magnification.

From Figure 5, it can be observed the morphology of the spruce biochar was used as filler in the development of the epoxy composites. From these pictures, it is clear that the microstructure of biochar was not affected by pyrolysis temperature. The HC prepared at 280 °C retained its original fiber skeleton and compact honeycomb spruce structure [40], which indicates that the thermal degradation of the wood did not occur during the hydrothermal carbon preparation process. From SEM images (Figure 5), it can be seen also that the epoxy materials possess a smooth surface, and the filler is well dispersed, the HC particles being evenly and uniformly spread throughout the resin. Likewise, with the increase of the filler percentage, one can remark the appearance of larger agglomerations of HC due to their high volume and surface (Figure 5e).

After a close investigation of the SEM images, it was observed that the pores of the HC particles were infiltrated with epoxy resin during the manufacturing process, thus leading to the creation of mechanical interlocking or physical bonding. Therefore, the RDGE resin not only embedded into the pores of HC but also held the biochar particles tightly as the binder, generating composites with good compatibility and adhesion between the polymeric matrix and the filler.

#### 3.3.5. Water Absorption

The moisture absorption of the prepared materials was evaluated using the ASTM D570 method. The water uptake evolution of the bio-based materials is displayed in Figure S6. According to reported studies, the water absorption of bio-based materials is influenced by a variety of factors [41,42]. One of the first defects that can influence the water absorption is generated by the presence of the micro-cracks and pores, which can appear in brittle matrices as in some epoxy resins. These cracks led to the moisture absorption in the neat resins, the saturation of the R95-BD5 resin being reached after 30 days absorbing around 0.79 wt %, while with the addition of HC the water absorption increases reaching up to 2.07 wt % for materials designed with 30 wt % hydrochar. The moisture uptake values are also directly correlated with the number of the free hydroxyl or other polar groups present in the biomaterial composition, which can increase the water absorption by hydrogen bonding with water [43,44].

#### 3.3.6. Solvent Stability

To determine their solvent stability, the materials were tested in various types of solvents such as MeOH, Ac, T, DMF, DMSO, AcN, and CHCl_3_ to cover a wide range of polar or apolar reagents. The sorption/degradation behavior of the bio-based materials is presented in Figure S8, while the physical appearance of the samples after 30 days of immersion is displayed in Figure S7.

Figure S8 shows the influence of solvents on the mass of materials, where a positive value implies an increase in weight and thus a swelling of the materials, while a negative value implies a reduction in weight, indicating the materials’ degradation. Based on these results, it can be said that all the composites have a relatively good stability in the tested solvents. After 30 days of immersion, only the neat resin lost ≈2–5 wt % in DMSO and AcN. In the case of MeOH, an increase in absorption occurs with the increase of the HC content, the maximum sorption value (4.86 wt %) being reached by the composite with 30 wt % HC. In addition, a small particle release, represented by solvent coloration in light brown, was observed for materials with more than 5 wt % HC in their composition. With increasing the HC amount in the materials’ composition, a more intense brown coloration was observed in DMF and DMSO. This brown coloration of the solvent may be due to the release of some unreacted HC particles, which then are displaced by the solvent, thus explaining the swelling of the tested materials. Only for toluene was no change in color found, and almost all the tested materials have a similar sorption of about 0.8 wt %. Considering these results, we can appreciate that the thermosets prepared with HC present a good resistance to the chemical reagents due to their high degree of crosslinking and stiffness.

## 4. Conclusions

This study investigated the possibility of replacing synthetic filler materials with a bio-based one, the hydrochar that is a product of hydrothermal carbonization of spruce bark wastes, to obtain sustainable biocomposites. Seven ratios of hydrochar were used along with epoxy resin in the preparation of biocomposites with better structural, thermal, and mechanical properties. A prior reactivity study was carried out to understand the influence of hydrochar on thermal curing and to establish the curing parameters. DSC analysis revealed that HC acted as coreactive, at the same time catalyzing the crosslinking process. This was obtained by increasing values normalized to the mass of the resin enthalpy of curing and also by decreasing the crosslinking temperature range, better defining the peak of reaction. FT-IR investigations of uncured mixture and cured products showed that during the crosslinking process, the epoxy ring was completely reacted, while possible chemical bonding between the HC and the polyether formed resin can be observed. The HC acted as a reactive filler. The SEM analysis images highlighted a very good interfacial adhesion between the epoxy matrix and the filler and a uniformly spread of HC particles throughout the resin with an occasionally small agglomeration of particles for a higher concentration of HC due to the higher volume and surface. The thermal properties of HC-based composites were improved by the addition of hydrochar: the thermosets resins present a very good thermal stability, losing 5 wt % of their mass at temperatures above 340 °C. The mechanical properties of bio-based composites investigated by DMA analysis, Shore hardness test, and tensile test showed that the hardness of the composite was improved from 82 SD for neat epoxy resin to 92 SD for HC composites, classifying these biocomposites as very hard materials.

## Figures and Tables

**Table 1:** Physico-chemical and mechanical properties of the bio-based thermoset materials.

Sample	T_5%_ (°C)	T_s_ (°C)	Hardness Test (SD)	Glass Transition (°C)				υ (mmol∙cm^−3^)	Density, g/cm^3^
				T_g_ (DSC)	T_g-onset_ (DMA)	T_g-loss_ (DMA)	Tan δ		
R95-BD5	337	190	82	101	82	93	99	8.23	0.9824
R94-BD5-HC1	339	189	85	101	80	89	97	8.89	1.2446
R90-BD5-HC5	347	192	89	100	81	90	98	14.55	1.2029
R85-BD5-HC10	339	190	86	102	81	92	98	15.90	1.2364
R80-BD5-HC15	344	194	92	96	76	84	95	14.29	1.1469
R75-BD5-HC20	345	191	92	99	78	86	94	15.39	1.0980
R65-BD5-HC30	340	191	89	98	73	84	89	20.50	1.0095

## Data Availability

The data presented in this study are available on request from the corresponding author.

## Supplementary Materials

The following are available online at https://www.mdpi.com/2073-4360/13/2/240/s1, Table S1: Physico-chemical characteristics of the epoxy resin components; Table S2 Particle size distribution of hydrochar; Table S3: Biocomposites’ formulations; Table S4: DSC results during heating and crosslinking of the neat resin and RDGE/BD/HC formulations; Table S5: Assignments of peaks absorptions identified in IR analysis for RDGE, BDMA and DMP-30; Table S6: Tensile properties of the bio-based polymeric materials; Figure S1: FT-IR spectra of hydrochar; Figure S2: FT-IR spectra of the raw materials; Figure S3: FT-IR spectra of unreacted and crosslinked composites; Figure S4: DTG curves of the bio-based materials; Figure S5: Stress-strain curves of the RD95-BD5, RD90-BD5-HC5, RD85-BD5-HC10, RD80-BD5-HC15, and R75-BD5-HC20; Figure S6: Water absorption as a function of immersed time; Figure S7: Solvent stability of thermosets; Figure S8: Solvent stability of the bio-based thermoset materials .
