# Supplementary material for: Hydrothermal Carbon as Reactive Fillers to Produce Sustainable Biocomposites with Aromatic Bio-Based Epoxy Resins
Source: Polymers (Basel). 2021 Jan 12;13(2):240. doi: 10.3390/polym13020240 (PMC7828177; doi:10.3390/polym13020240)
Supplement: Supplementary file 1 [file polymers-13-00240-s001.pdf]

polymers

MDPI

Supporting Information

Hydrothermal carbon as reactive fillers to produce sustainable biocomposites with aromatic biobased epoxy resins

Iuliana Bejenari,[a,b] Roxana Dinu,[a] Sarah Montes,[c] Irina Volf,[b] Alice Mija[a]*

[a]University Côte d'Azur, Institute of Chemistry of Nice, UMR CNRS 7272, 06108 Nice Cedex 02, France

[b]Gheorghe Asachi Technical University of Iasi, Faculty of Chemical Engineering and Environmental Protection, 73 Prof. D. Mangeron Street, 700050, Iasi, Romania

[c]CIDETEC, Basque Research and Technology Alliance (BRTA), Po. Miramón 196, 20014 Donostia-San Sebastián, Spain

*E-mail : Alice.Mija@unice.fr

Total number of pages : 8

Total number of tables : 6

Total number of figures : 8

1. Compounds characteristics

Table S1. Physico-chemical characteristics of the epoxy resin components

Compound Name	Molecular structure	Molar mass (g/mol)	Appearance	Density (g/mL at 25°C)
Resorcinol diglycidyl ether (RDGE)	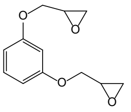	222.24	White to Light Yellow Semi-Solid to Solid	1.21
N, N-Dimethylbenzylamine (BDMA)	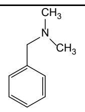	135.21	Colorless Liquid	0.9

Copyright: © 2021 by the authors. Licensee MDPI, Basel, Switzerland. This article is an open access article distributed under the terms and conditions of the Creative Commons Attribution (CC BY) license (http://creativecommons.org/licenses/by/4.0/).

2,4,6-Tris(dimethylaminomethyl)phenol (DMP-30)	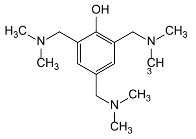	265.39	Pale yellow Viscous liquid	0.969

Table S2. Particle size distribution of hydrochar

Particle diameter, µm	Density distribution, %
0.1-0.4	0.05
2-6	0.09
6-20	0.15
20-50	2.5
80-200	3.5
300-500	15

Table S3. Biocomposites' formulations

Samples abbre-viation	Compounds ratio in biocomposites, in wt.%			
	Resorcinol diglycidyl ether (R)	Hydrochar (HC)	N,N-dimethylbenzylamine (B)	2,4,6-tris(dimethylaminomethyl)phenol (D)
R95-BD5	95	0	2.5	2.5
R94-BD5-HC1	94	1	2.5	2.5
R90-BD5-HC5	90	5	2.5	2.5
R85-BD5-HC10	85	10	2.5	2.5
R80-BD5-HC15	80	15	2.5	2.5
R75-BD5-HC20	75	20	2.5	2.5
R65-BD5-HC30	65	30	2.5	2.5

2. Differential scanning calorimetry

Table S4. DSC results during heating and crosslinking of the neat resin and RDGE/BD/HC formulations

Sample code	Reaction T_{max} (interval of reaction) (°C)	Enthalpy of reac-tion ($J.g^{-1}$)
R95-BD5	141 (66-229)	405
R94-BD5-HC1	137 (63-215)	440
R90-BD5-HC5	130 (60-215)	478
R85-BD5-HC10	121 (55-207)	511
R80-BD5-HC15	120 (56-201)	450
R75-BD5-HC20	116 (53-188)	503
R65-BD5-HC30	112 (52-172)	512

3. FT-IR Spectroscopy

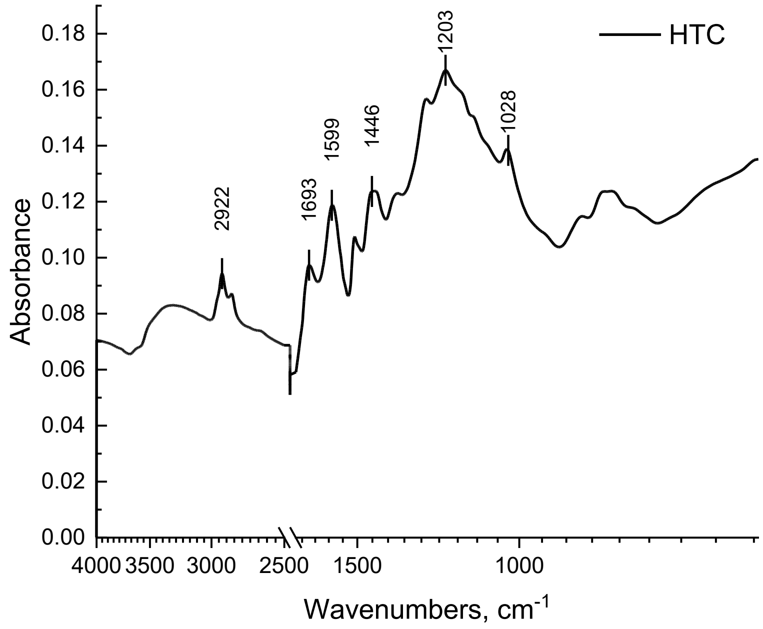

Figure S1. FT-IR spectra of hydrochar

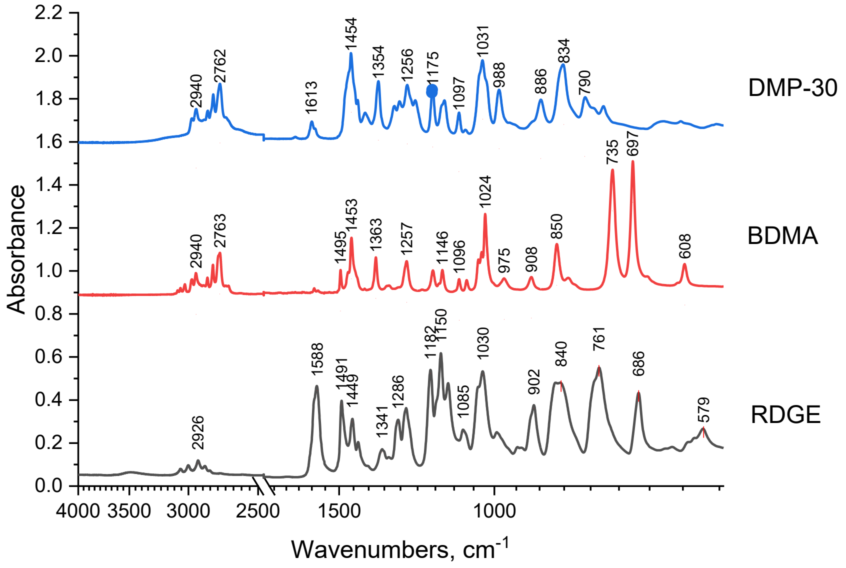

Figure S2. FT-IR spectra of the raw materials

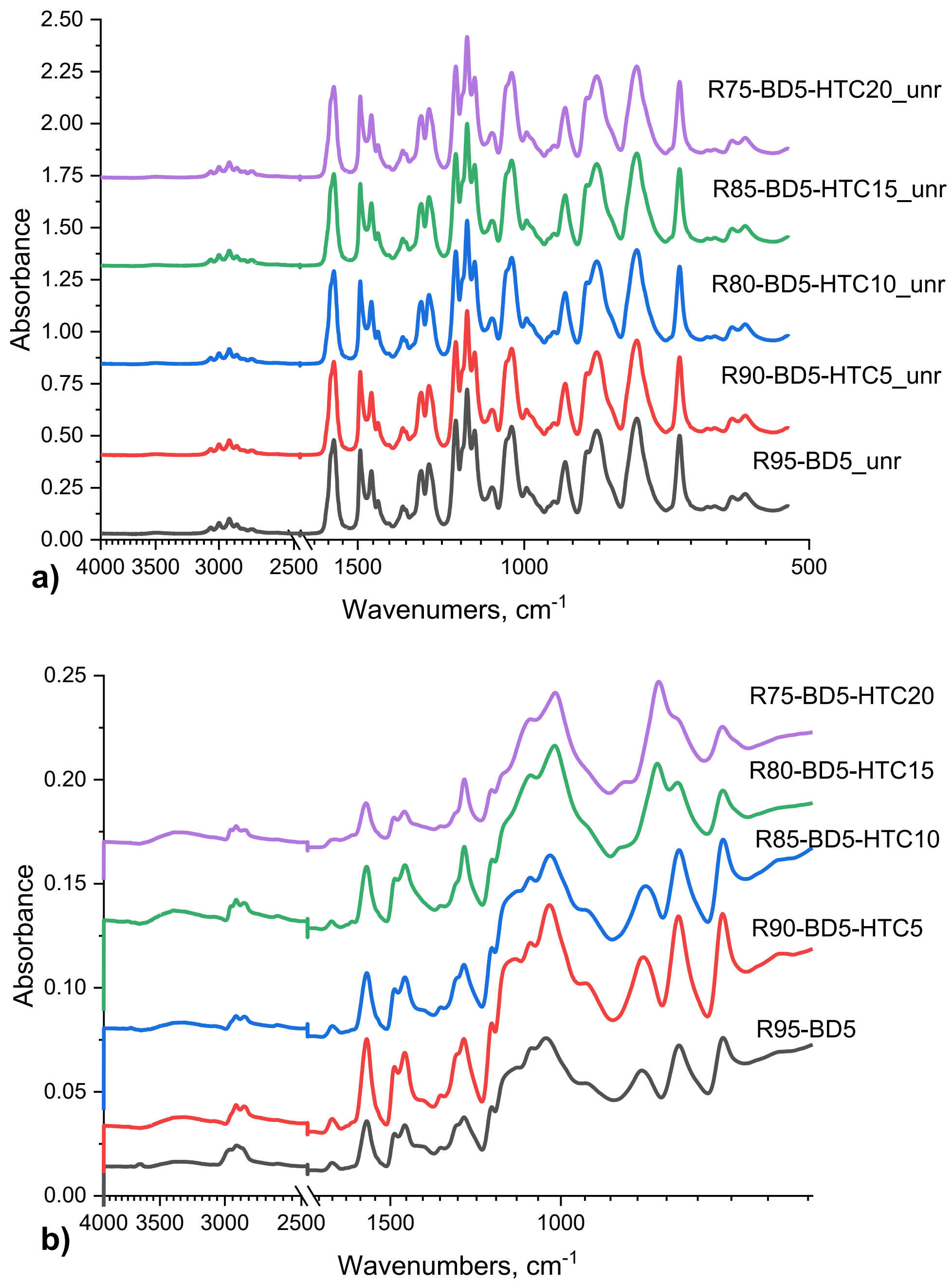

Figure S3. FT-IR spectra of unreacted and crosslinked composites

Table S5. Assignments of peaks absorptions identified in IR analysis for RDGE, BDMA and DMP-30

Compounds	Wavenumbers, cm^{-1}	Functional groups
RDGE	3485	O-H stretching of hydroxyl groups
	3062	Stretching of asymmetrical C-H bond in aromatics
	2924, 2873	Symmetrical and asymmetrical stretching of C-H aromatic and aliphatic rings
	1590	Stretching vibration of C=C bond of aromatic ring
	1491, 1449, 1427	Stretching vibration of C-C bond of aromatic ring
	1340	Stretching bond deformation of C-H in plane
	1286, 1259	epoxide ring vibration
	1182, 1150, 1129, 1085, 1030	Stretching vibration of asymmetrical C-O-C bond of

		ethers
	902	Stretching vibration of C-O bond of oxirane group
	842	Stretching vibration of C-O-C bond of oxirane group
	761, 686, 580	Stretching vibration of C-H out of plane deformation in aromatic
BDMA	3085, 3063, 3027, 2973, 2941, 2854, 2814, 2763	Asymmetrical and symmetrical stretching vibration of C-H bond of $–CH_3$ and $–CH_2$ groups
	1495, 1452, 1363, 1317	Asymmetrical and symmetrical stretching deformation of C-H bond of $–CH_3$ and $–CH_2$ groups
	1258, 1174, 1146, 1097, 1075, 1034	Stretching vibration of C-N bonds corresponding to amine groups
	975, 908, 850, 825, 735, 697, 609	Out-of-plane deformation of =CH bonds
DMP-30	2973, 2940, 2853, 2811, 2763,	Asymmetrical and symmetrical stretching vibration of C-H bond of $–CH_3$ and $–CH_2$ groups
	1683, 1611	Stretching vibration of C=C skeleton in benzene ring
	1455, 1402, 1354	Asymmetrical and symmetrical stretching deformation of C-H bond of $–CH_3$ and $–CH_2$ groups
	1299, 1281, 1256, 1230, 1032	Stretching vibration of C-N in amine
	1175, 1139, 1097	Stretching vibration of C-O in phenol
	988, 886, 836, 789, 752, 643, 615	Out-of-plane deformation of =CH bonds

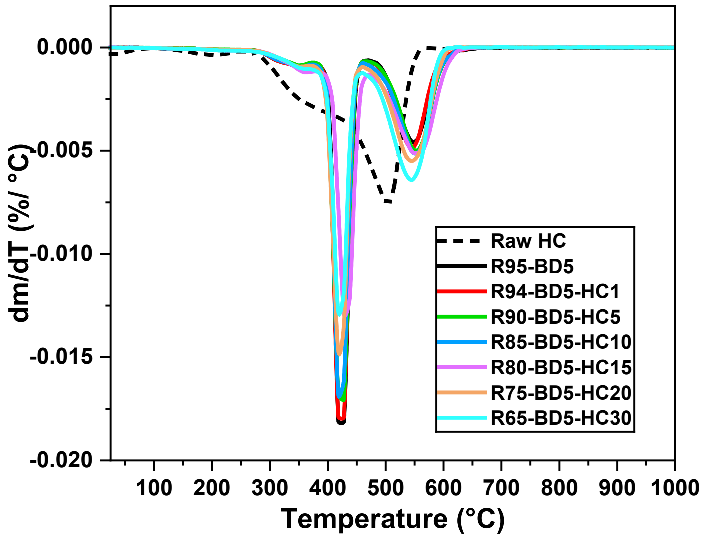

Figure S4. DTG curves of the bio-based materials

4. Thermogravimetric analysis

5. Tensile testing

Table S6. Tensile properties of the bio-based polymeric materials

	Young's Modulus (MPa)	Maximum Stress (MPa)	Stress at Failure (MPa)	Maximum Strain (%)	Strain at Failure (%)	Resilience (MJ/m^{-3})
**R95-BD5**	1796.28 ± 38.41	63.41 ± 9.38	57.71 ± 12.48	6.84 ± 2.36	6.86 ± 2.36	216.9

**R90-BD5-HC5**	1731.14 ± 88.61	24.80 ± 7.62	24.80 ± 7.62	1.86 ± 0.72	1.86 ± 0.72	23.1
**R85-BD5-HC10**	1572.60 ± 55.57	22.84 ± 6.96	22.84 ± 6.96	1.60 ± 0.48	1.60 ± 0.48	18.3
**R80-BD5-HC15**	1740.94 ± 414.49	19.68 ± 0.85	19.68 ± 0.85	1.34 ± 0.24	1.34 ± 0.24	13.2
**R75-BD5-HC20**	1398.16 ± 160.57	17.58 ± 3.84	17.58 ± 3.84	1.46 ± 0.19	1.46 ± 0.19	12.8

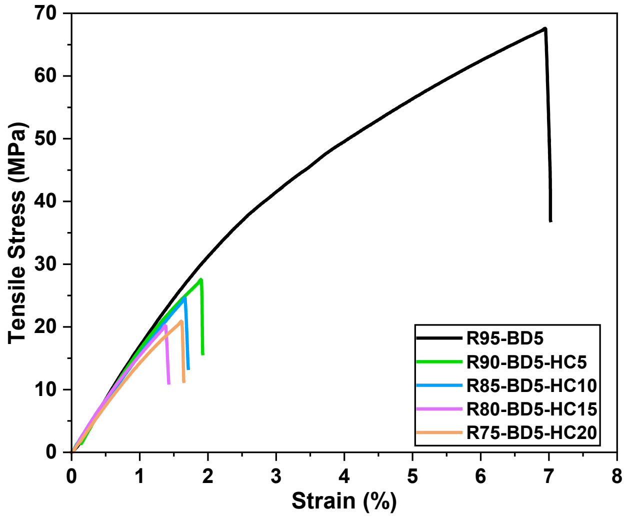

Figure S5. Stress-strain curves of the RD95-BD5 (black line), RD90-BD5-HC5 (green line), RD85-BD5-HC10 (blue line), RD80-BD5-HC15 (purple line), and R75-BD5-HC20 (orange line)

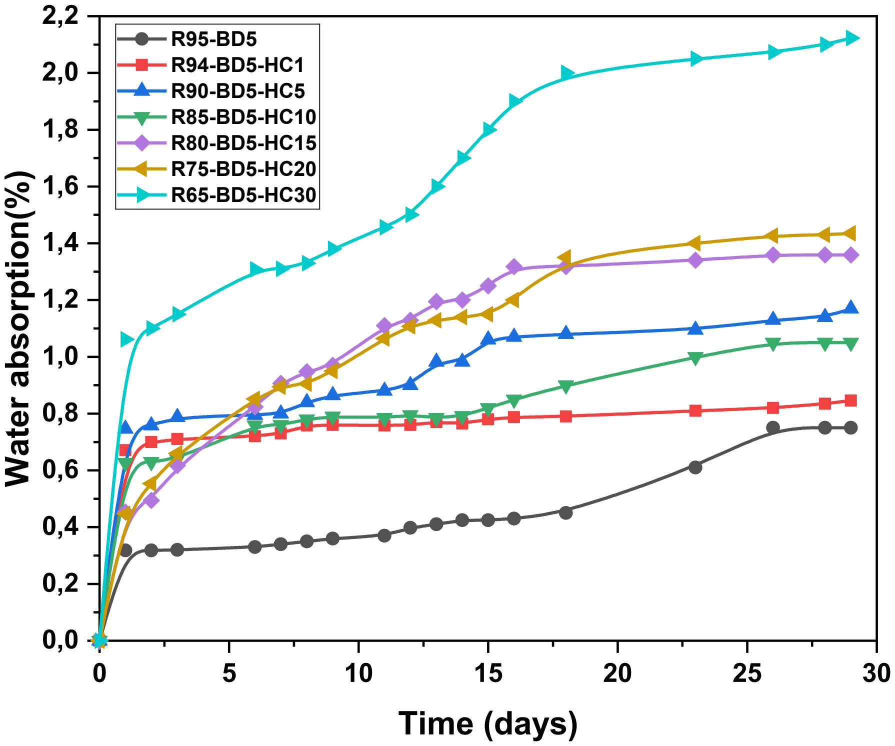

Figure S6. Water absorption as a function of immersed time

6. Water absorption

7. Solvent stability

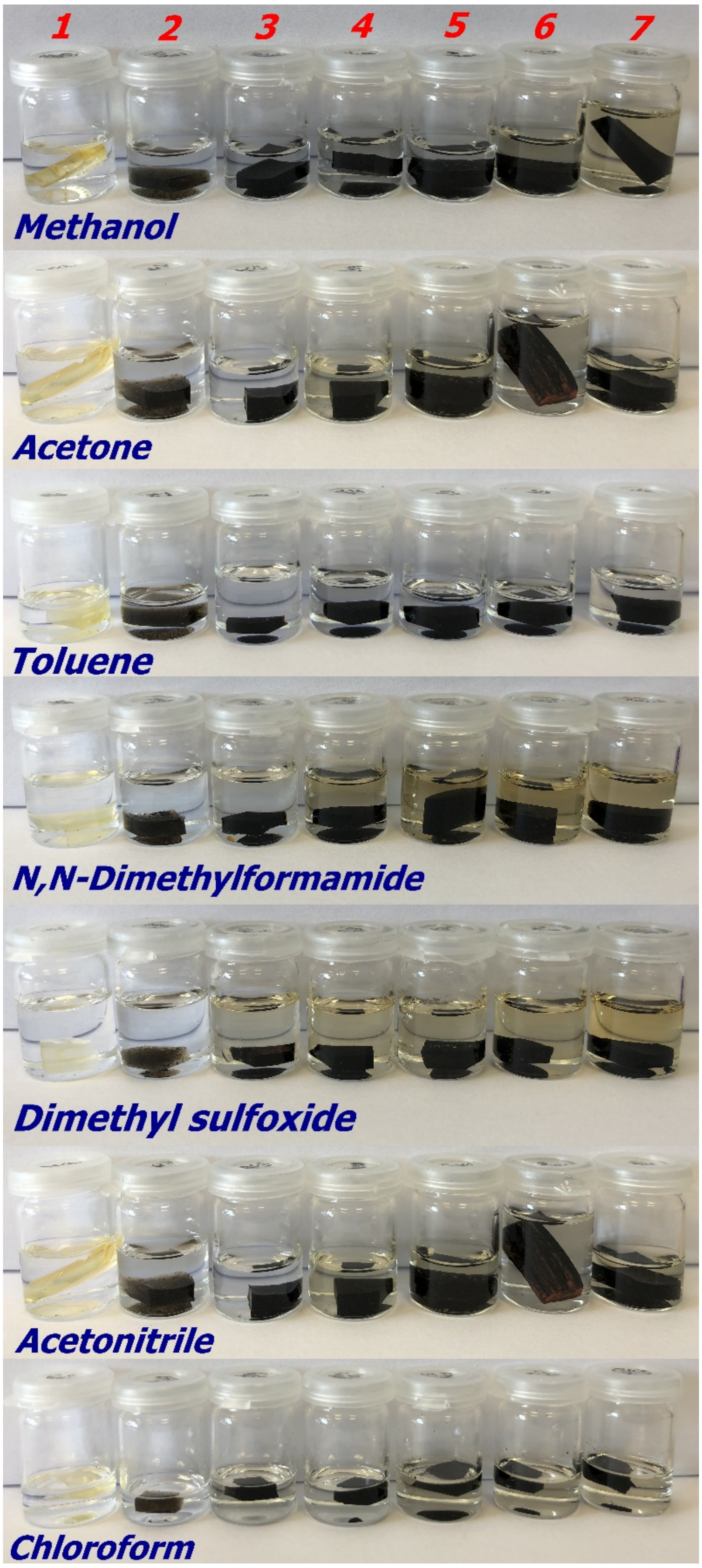

Figure S7. Solvent stability of thermosets: 1) R95-B5; 2) R94-BD5-HTC1; 3) R90-BD5-HTC5; 4) R85-BD5-HTC10; 5) R80-BD5-HTC15; 6) R75-BD5-HTC20; 7) R65-BD5-HTC30

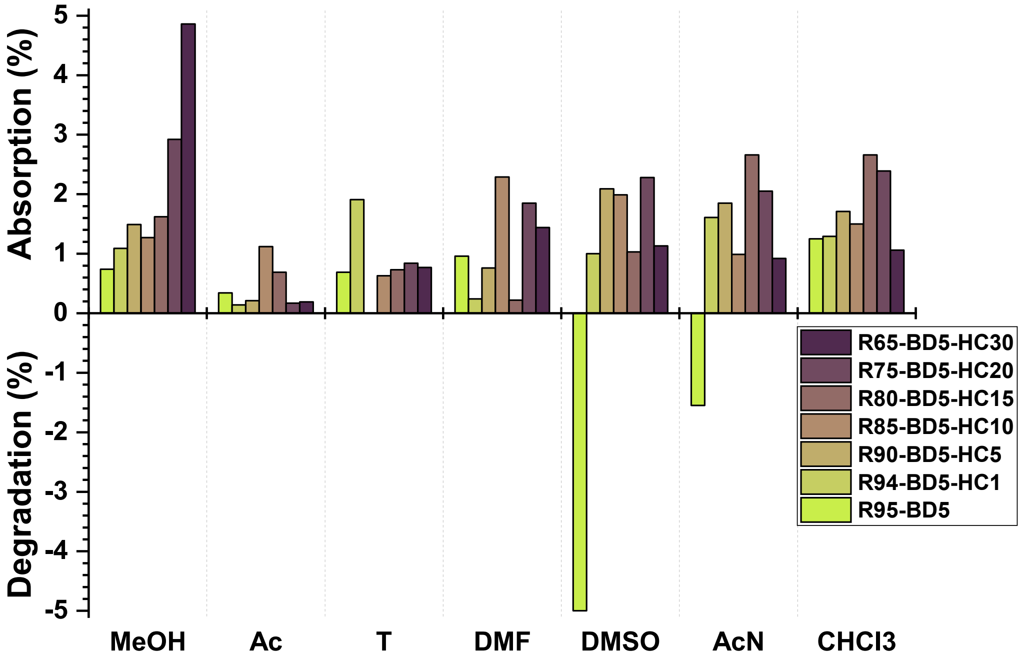

Figure S8. Solvent stability of the bio-based thermoset materials
